# Atypical chemokine receptors

**DOI:** 10.1016/j.yexcr.2011.01.012

**Published:** 2011-03-10

**Authors:** Maria Helena Ulvmar, Elin Hub, Antal Rot

**Affiliations:** MRC Centre for Immune Regulation, University of Birmingham, Edgbaston, Birmingham, B15 2TT, UK

**Keywords:** ACR, DARC, D6, CXCR7, CCRL1, CCRL2, Interceptor

## Abstract

Atypical chemokine receptors (ACRs) are cell surface receptors with seven transmembrane domains structurally homologous to chemokine G-protein coupled receptors (GPCRs). However, upon ligation by cognate chemokines, ACRs fail to induce classical signaling and downstream cellular responses characteristic for GPCRs. Despite this, by affecting chemokine availability and function, ACRs impact on a multitude of pathophysiological events and have emerged as important molecular players in health and disease. This review discusses individual characteristics of the currently known ACRs, highlights their similarities and differences and attempts to establish their group identity. It summarizes the progress made in mapping ACR expression, understanding their diverse *in vitro* and *in vivo* functions of ACRs and uncovering their contributions to disease pathogeneses.

## Introduction

Directed movement of cells defines embryogenesis and importantly contributes throughout life to many physiological and pathological processes. Among different molecules able to provide directional cues and drive augmented-rate cell locomotion, chemokines occupy a unique position [1]. The unparalleled molecular diversity of these structurally homologous chemotactic molecules [2] and the fact that chemokines act in congruent manner, allows them to convey most diverse and subtle cellular messages and orchestrate most intricate cellular moves [1]. Human chemokines are the products of 48 distinct genes. However, due to existing polymorphisms, differential splicing, N- and C-terminal enzymatic processing and also accounting for the virally encoded chemokines, the number of functional chemokine entities acting in humans well exceeds hundred different molecules [3,4]. Chemokines transmit their signals through one or more of 18 distinct heptahelical G-protein coupled receptors (GPCRs), which can be triggered each by either one or up to ten different chemokines, albeit with disparate potency, efficacy and texture of resulting downstream cell responses [1]. Most chemokines are uniquely recognized, if not by one, but by a set of chemokine GPCRs. Therefore, despite enormous diversity, there is little if any functional redundancy within the chemokine system [1]. The emergence of alternative, “atypical” chemokine receptors (ACRs) increased even further the interactive complexity within the chemokine system [5,6]. ACRs are heptaspanning membrane receptors homologous to chemokine GPCRs. But, because of the modified or missing canonical DRYLAIV motif within the second intracellular loop, they are unable to couple to G-proteins and fail to induce the full spectrum of “classical” GPCR signaling and cellular responses, including cell migration [5,6]. This led to their exclusion, in part, from the systematic chemokine receptor nomenclature and initial designation as “silent”. It is clear now that ACRs can signal with their downstream biochemical cascades corresponding to G-protein-independent signaling of GPCRs [7–9]. An important GPCR feature retained by ACRs is their ability to efficiently internalize their chemokine ligands, hence they are also called ‘interceptors’ (*inter*nalizing re*ceptors*) [5,10]. Currently the ACR family comprises five receptors, Duffy Antigen Receptor for Chemokines (DARC), D6, CXCR7 and CC-Chemokine Receptors like 1 and 2 (CCRL1 and CCRL2), which cover among them a wide range of chemokine ligands. In general, the ACR-mediated internalization and subcellular localization of chemokines and the ultimate fate of cognate ligands falls between two diametrically opposite paradigmatic outcomes. On one side of the spectrum is the lysosomal targeting and degradation of chemokines, the reason for the popular designation of ACRs as scavenging “decoys” [6]. This ACR function is represented most emphatically by D6 [11]. On the opposite side is the transcytosis function allowing ACRs to transport chemokines across biological barriers and concentrate them in hard-to-reach microanatomical domains. This ACR function is currently epitomized by DARC [12]. However, data on the outcomes of chemokine interactions with ACRs are derived almost entirely from *in vitro* studies in heterologous transfectants. Therefore, true subcellular functions of ACRs may be different *in vivo* and may vary depending on the cellular context.

Curiously, irrespective of either scavenging or transporting chemokines, ACR activity may lead to the establishment of chemokine patterns, which would not be able to form as a result of the free diffusion of chemokines. Such functional chemokine patterns, either soluble or immobilized [13], often referred to as gradients, may be responsible in several *in vivo* settings for the directionality of cell responses to chemokines. Therefore, even those ACRs, which contribute to chemokine degradation, may support directional chemokine-induced *in vivo* cell migration. In addition to affecting chemokine positioning within tissues, ACRs may influence the responses to chemokines by heterodimerizing with chemokine GPCRs expressed in cis-geometry, thus modifying their availability and signaling. ACRs may also interfere with or consume secondary intracellular signaling molecules, β-arrestin in particular. Such mechanism characterizes the function of C5L2, an interceptor for chemoattractant C5a [14].

Chemokine GPCRs may also exhibit the full spectrum of features characteristic of ACRs. In some settings chemokine GPCRs are uncoupled from G-proteins and downstream signaling e.g. in response to physiological or pharmacological stimuli [15], in cell senescence [16], or when expressed in particular cells [17]. Such G-protein-“uncoupled” receptors were shown to scavenge or transport chemokines [17,18]. However, the removal of cognate chemokines from tissues is also an important, but often overlooked, function of signaling chemokine GPCRs, as convincingly exemplified by CCR2 [19]. Nevertheless, it is not GPCRs, but specialized chemokine interceptors, the ACRs, which are positioned best to regulate chemokine availability and create functional chemokine patterns within tissue microenvironments. The highly conserved structure of ACR orthologues and the co-evolvement of ACRs simultaneously with chemokine GPCRs (both appeared in jawless fish ca. 650 million years ago [20]) indicate an important non-redundant nature of ACRs and the requirement of both of these receptor types for optimal chemokine function *in vivo*.

Individual expression patterns of ACRs, also diverse ACR activities, including various outcomes of their interactions with chemokines and how these impact on chemokine functions, are discussed in some detail below.

## DARC, a blood group antigen

DARC was initially described as the Duffy (Fy) blood group antigen [21]. It consists of two major co-dominant alleles, FY*A and FY*B, which constitute one of 26 currently recognized blood group systems. The antigen was named after Mr. Duffy, a hemophiliac first shown to develop antibodies to Fya+ erythrocytes [21]. The Fya+ phenotype was described one year later in Mrs. Hahn, a multipara with anti-Fyb titers who had been exposed to fetal Fyb+ erythrocytes during pregnancies [22]. The two Fy alleles differ by a single base substitution 306 G→A in codon 44 encoding glycine in Fya and aspartic acid in Fyb [23]. In addition to Duffy “positive” homozygous and heterozygous phenotypes, there is a remarkable Duffy “negative” phenotype carried by the majority of individuals of West African ancestry. It is determined by a single T to C substitution at nucleotide − 46 within the binding region of the erythroid GATA1 promoter of FY*B [24]. This polymorphic change terminates transcription of DARC in erythroid cells only [24] and represents the third major allele, the erythroid silent FY*B(ES). Therefore, Duffy “negatives” still bear DARC at non-erythroid sites of its expression, on venular endothelial cells [25], Purkinje neurons [26] and epithelial cells of kidneys and lungs [27].

Two malarial parasites, *Plasmodium vivax* and *knowlesi* use DARC to invade erythrocytes [28,29]. It was suggested that the Duffy negative phenotype evolved to protect its carriers from vivax malaria [28,29], which, accordingly, occurs seldom in Africa. Nevertheless, recent epidemiological evidence suggests that such resistance is not absolute. Some Duffy negative individuals on Madagascar, inhabited by a mixed Duffy positive and negative indigenous population, suffer from symptomatic vivax malaria and carry parasites asymptomatically [30]. These findings suggest that the failure of Duffy negative individuals to infect the anopheles vector by vivax parasites may be an important component of the resistance to malaria by a population as a whole. Another rare FY*A null mutation developed in Plasmodium vivax-endemic region of Papua New Guinea causing two-fold depletion of Fya expression and protection against vivax malaria [31]. Conversely to vivax, *Plasmodium falciparum* does not require DARC for erythrocyte invasion and therefore the Duffy negative phenotype does not protect its bearers from falciparum malaria. A rare C286T substitution in FY*B (FYX allele) leads to a “weak” Fyb expression [32] while other rare polymorphisms cause a Duffy negative phenotype [33–35].

HIV-1 also binds to DARC on erythrocytes allowing them to transmit the virus to mononuclear and other susceptible blood cells [36,37]. This mechanism of trans-infection may explain why Duffy negative individuals, despite having a higher risk of acquiring HIV, exhibit a slower disease progression [36], especially when comparing the subgroups of leukopenic patients [38]. The impact of Duffy negative phenotype on HIV infection has been intangible in some cohorts [39–43].

## Duffy antigen, receptor for chemokines

The observation that erythrocytes of Duffy-positive but not negative individuals bind IL-8 led to the establishment of Duffy antigen as a chemokine receptor [44–46]. DARC binds, albeit with a broad range of affinities, 20 human inflammatory chemokines of both CC and CXC families [44–48]. Initial cloning suggested that DARC has nine transmembrane domains [49] and only later it was modeled as a seven-spanner [50]. Characteristically, DARC lacks completely the GPCR consensus motif DRYLAIV and fails to couple to G-proteins and trigger classical GPCR signaling. Red blood cells are considered incapable of endocytosis. Therefore, chemokines associated with erythrocyte DARC remain on the cell surface and potentially can be eluted by cognate chemokines or other molecules, e.g. heparin or activated coagulation factors [51]. Conversely, when expressed in nucleated cells, DARC can efficiently internalize cognate chemokines, a characteristic interceptor feature of ACRs [5]. In contrast to some ACRs, which in the absence of ligands continuously recycle between the cell membrane and intracellular vesicular compartments [52,53], DARC internalization is induced by its ligation [12,54]. This chemokine-induced response signifies a signaling event of yet unknown molecular nature, which may involve β-arrestin, Rab GTPases and kinases shown to be activated in GPCRs independently of G proteins [7–9]. In contrast to D6, CXCR7 and CCRL1, all more or less efficiently scavenge their cognate ligands [11,53,55,56], chemokine internalization by DARC does not lead to their degradation. Nevertheless, DARC-mediated chemokine endocytosis may remove chemokines from extracellular microenvironments. Such depletion of extravascular chemokines by the endothelial cell DARC may provide the mechanism how it negatively regulates angiogenesis induced by ELR chemokines [57] and in the context of tumors [58–60]. Alternatively, DARC can heterodimerize with chemokine GPCRs and thus affect their responses to cognate chemokines [61].

In addition to chemokines, DARC was suggested to bind in trans-geometry CD82, a tetraspanin expressed by tumor cells [62]. Through this interaction DARC apparently inhibits tumor cell proliferation and induces senescence [62]. Currently, it is not clear if a direct molecular interaction between DARC and CD82 takes place or CD82 is required for the organization of functional membrane domains, which include molecules interacting with DARC. Also, this study contains a conundrum. Human umbilical vein endothelial cells were used as a source of DARC. This cell type is normally completely devoid of DARC, *in situ* and *in vitro*.

## Erythrocyte DARC regulates chemokine homeostasis

In concert with early ideas about the function of ACRs, it has been assumed initially that the role of DARC is limited to negative regulation of chemokine activities. This was concluded based on the chemokine “sink” function ascribed to the erythrocyte DARC [44] and corroborated further when DARC was shown to mitigate pathological surges of circulating inflammatory chemokines and dampen the ensuing systemic leukocyte activation [63]. Curiously, by acting as a sink and protecting circulating leukocytes from chemokine overstimulation and desensitization, erythrocyte DARC can preserve leukocyte responsiveness to tissue-derived localized pro-emigratory cues and enhance leukocyte emigration from blood. However, systemic pre-exposure to chemokines may, on the contrary, also prime leukocytes for a multitude of effector functions [64,65], including enhanced chemokine-induced migration [66]. This means that erythrocyte DARC, by reducing plasma chemokine levels, can also mitigate subsequent leukocyte responses. These two diametrically opposite possible outcomes may contribute to the complex and apparently conflicting phenotypes of leukocyte recruitment observed in DARC deficient mice [63,67–71]. The formula of DARC involvement is further complicated by its ability in both humans [72] and mice [73] to act as a chemokine depot and sustaining chemokine levels not only on erythrocytes but also in plasma. To date it is not clear what purpose is served by maintaining inflammatory chemokines in blood, but it may have functional consequences [68]. Chemokines, depending on their relative binding affinities for DARC, can shift the levels of other erythrocyte-borne and free blood chemokines with ensuing effects for leukocyte responses [68]. In a mouse model of *E. coli* pneumonia the overproduction of CXCL5, a chemokine with high affinity for DARC, resulted in saturation of erythrocyte DARC and inhibition of CXCL1 and CXCL2 binding [68]. The increase in plasma levels of these chemokines caused desensitization of the cognate receptor CXCR2 on neutrophils and, as a consequence, decreased migratory responses.

Surprisingly little is known on how distinct characteristics of chemokine homeostasis in Duffy-negative individuals impact on the development of human diseases. Nevertheless, there are correlates of Duffy negative polymorphism with incidences of benign ethnic neutropenia [74] and asthma and high IgE [75]. Recently, remarkable difference in the abilities of human polymorphic DARC variants Fya and Fyb to maintain chemokine plasma levels have been uncovered in genome-wide association studies [51]. Mechanistically, it is not clear if this results from differential chemokine affinities of polymorphic DARC variants or for other reasons, e.g. levels of DARC expression.

## DARC function in endothelial cells

Leukocyte egress from blood occurs predominantly in venules and small veins where chemokines can be immobilized on the luminal membrane of endothelial cells [76] by glycosaminoglycans (GAGs), heparan sulfate in particular [77,78]. In order to appear on the luminal side of the endothelium, tissue-derived chemokines have to cross the endothelial cell barrier. This is achieved by active transcellular transport targeting chemokines to the apical microvilli [79]. Heparan sulfate was shown to mediate chemokine transcytosis [80] but it was also suggested that DARC, expressed by venular endothelial cells [25,27,81] may participate in chemokine binding and transport [25,82,83]. Recent *in vitro* studies unequivocally established DARC as a transcytosis receptor that transports chemokines unidirectionally, from the baso-lateral to apical side only, but not *vice versa* [12]. Accordingly, DARC expression supports optimal chemokine-induced leukocyte migration *in vitro* and *in vivo* [12]. Upregulation of DARC expression in veins and its appearance in vascular beds usually devoid of it, was observed in infection, inflammation and transplant rejection [84–90]. Though, it is not clear if DARC overexpression in these lesions is required for their development. Chemokine transcytosis by DARC across venular endothelial cells also may provide a mechanism of chemokine elimination from the tissues. Clearance of chemokines, however, in the overwhelming majority of tissues, is more likely to take place by diffusion between the endothelial cells of blood capillaries or via the lymphatics. The initial afferent lymphatics are completely devoid of DARC-immunoreactivity. Conversely, a segment of lymphatic vessels, the podoplanin-dull precollectors express DARC, suggesting its involvement in chemokine mediated cell migration at this site [91]. It is unknown under what circumstances, in response to which chemokines and by what mechanism such trafficking may take place.

## D6, a CC-chemokine scavenger

D6 is an ACR for at least twelve inflammatory CC chemokines and has a high homology with their classical GPCRs [92]. Consistently with being an ACR, D6 has DKYLEIV instead of the canonical DRYLAIV motif. Therefore, no G-protein-mediated signaling is initiated downstream of D6. But, remarkably, D6 may be not as “silent”, as it has always been postulated but a permanently “chitchatting” receptor. It was revealed that D6 constantly undergoes phosphorylation at its C-terminus [93,94] putting it on a par with continuously signaling virally encoded receptors KSHV-GPCR and US28 [95,96]. It is of note that phosphorylation of D6 was not observed in all studies [52]. However, it is unequivocal that D6 is internalized, recycled and re-expressed constitutively, in either presence or absence of cognate ligands [52,53,97]. Moreover, chemokine uptake not only fails to downmodulate D6 or desensitize it for subsequent chemokine binding, but actually enhances the cell surface expression of D6 [53,97]. This is due to translocation of D6 from intracellular rab4-, rab11- and transferrin receptor-positive early and recycling endosomes, where it localizes under basal conditions [52,53,97]. These are the intracellular compartments where the chemokine cargo also initially appears following its internalization by D6. Subsequent transfer into late endosomes leads to dissociation of chemokines and their degradation in lysosomes, whereas D6 is recycled back to the cell surface. Repeating this cycle allows D6 to massively scavenge CC chemokines [11,52,53,97]. The residues within the C-terminus of D6 determine its intracellular itinerary and also the resistance to desensitization but, not the internalization itself [94].

A characteristic feature of almost all D6 ligands is a proline in N-penultimate position [98]. However, this residue is required only for chemokine degradation initiated by D6, but not for their binding to D6 [99]. Using full length inactive “precursor” chemokine CCL14(1–74) and its N-terminally processed forms, active CCL14(9–74) and CD26-cleaved inactive CCL14(11–74), it was shown that, whereas all three molecules bind D6, only the active agonist CCL14(9–74) is degraded and can induce the upregulation of D6 expression. Similar observations were made for D6-degraded CCL3L1, poorly degraded CCL3 and non-degraded CCL3(5–68), all binding to D6 [99]. This experimental work has profound implications for understanding the function of D6. It shows that chemokine binding to D6 can be dissociated from downstream events of ligand-induced upregulation of D6 surface expression and targeting chemokines for lysosomal degradation. Together with previous findings [94], this implies that chemokines signal through D6, with ligand-induced receptor upregulation being the main cellular effect [99]. Proline residue in penultimate N-terminal position of chemokines [99] and the serine-rich C terminal tail of D6 [94] are required, but the molecules involved in downstream signaling remain elusive. It is also not clear what is the fate of those D6 ligands, which are not degraded as a consequence of their binding.

## D6 expression and function *in vivo*

The mapping of D6 mRNA expression in human and mouse tissues revealed its presence in many organs and tissues including lung, liver, spleen, kidney, heart, muscle, brain, placenta, gut and skin [92,100]. Immunohistochemistry and *in situ* hybridization showed that in several of these tissues D6 is expressed by the endothelial cells lining afferent lymphatic vessels [101] whereas *in situ* chemokine binding studies [102] suggested that D6 in these cells is functional [82,101]. However, it is not known what is the mechanistic role of D6 at this site of intense cell traffic and fluid diffusion. Tissue-derived chemokines, like other small molecules, are swept through the lymphatics into the draining lymph nodes where they can be loaded onto the endothelial cells of high endothelial venules and induce leukocyte homing into the lymph nodes [103,104]. D6 expressed on the luminal side of the lymphatic endothelium may scavenge chemokines during their passage along the lymphatics. This would limit the “remote control” function of tissue chemokines in the lymph node. Also, it is possible that chemokine neutralization by D6 prevents leukocyte activation and consequently adhesion to the endothelial lining of lymphatic vessels promoting their channeling into the lymph nodes. Alternatively, by scavenging chemokines D6 on lymphatics may lead to the establishment of their putative gradients, which either induce leukocyte entry into the lymphatics or prevent leukocyte return back into the tissue. Additionally, D6 is expressed by several leukocyte subtypes, B-cells, dendritic cells, macrophages and monocyte subsets [105]. Chemokine scavenging by leukocyte D6 may provide the most efficient way to reduce the chemokine contents within the inflammatory lesions and thus contribute to the resolution of inflammatory pathologies [105,106]. Pro- and anti-inflammatory mediators regulate the D6 levels on leukocytes leading to teleologically consistent ensuing effects. *In vitro* stimulation of monocytes and macrophages with major pro-inflammatory cytokines leads to a decrease in D6 expression, whereas treatment with TGFβ results in its increase [105,107]. Because chemokine GPCRs and ACRs can heterodimerize [61,108] it is also possible that D6 expressed by leukocytes directly influences chemokine-induced leukocyte responses mediated via their GPCRs.

## Pathophysiological role of D6: lessons from D6 deficient mice

The use of D6 knockout mice enabled an array of *in vivo* studies probing the function of this ACR in different diseases. Under normal conditions D6 deficiency does not convey any apparent disadvantages. However, when challenged with various stimuli D6 knockout mice develop increased inflammatory responses. For example, following topical treatment with phorbol ester or Freund's adjuvant injection D6^−/−^ mice show augmented skin inflammation as compared to wild type controls [109,110]. Also, in two-step chemical skin carcinogenesis model, D6 expression suppressed tumor development by scavenging chemokines and mitigating the recruitment of tumor-promoting inflammatory cells [111]. There are other examples of a protective function of D6 in murine pathologies. In experimental *Mycobacterium tuberculosis* infection D6 expression prevented the lethal outcome seen in D6 knockouts by suppressing the chemokine and cytokine storm and reducing leukocyte infiltration into the lungs and other organs [112]. A similar mechanism of action by D6 was implicated in a protective effect in a model of carbon-tetrachloride induced liver injury [113].

In placenta, an organ with the highest D6 expression, it is on syncytiotrophoblasts, cells covering placental villi [114,115]. Additionally, decidua and gestational membranes express D6 abundantly throughout the human pregnancy [115]. It is easy to envision how a chemokine scavenging receptor placed at the foeto-maternal interface would protect the fetus from maternal chemokines. To investigate this, two types of pathologies leading to fetal loss in humans were modeled in D6 deficient mice. On one hand, it was revealed that placental inflammation induced by LPS or anti-phospholipid antibodies was more massive in D6 knockout mice and resulted in a significant increase in fetal loss as compared to wild type controls [114]. On the other hand, placental D6 was shown to ward off maternal immune assault against an allogeneic fetus [115]. D6 deficient embryos transferred into allogeneic surrogate mothers were resorbed and showed abnormalities more often than their wild type counterparts [115].

However, not in all experimental models D6 plays an unequivocally protective role. D6 is expressed in the brain, primarily by astrocytes [116], ideally positioned to scavenge pathogenic inflammatory chemokines. Yet, D6 knockout mice are relatively protected from experimental autoimmune encephalitis induced by myelin oligodendroglial glycoprotein immunization [117]. This is explained by a reduced aptitude of D6 deficient mice to generate adaptive immune responses due to a failure of dendritic cell trafficking through the lymphatics [117]. D6 is expressed in murine colon and upregulated in colitis induced by dextran sulfate sodium (DSS) [118]. Nevertheless, the overall pathology of DSS-colitis was significantly reduced in D6 deficient mice as compared to wild type controls, though accompanied by increased IL-17A and IFNγ production and elevated numbers of Tγδ cells [118]. Another report showed in a similar model that D6 deficient mice have increased levels of chemokines, enhanced recruitment of leukocytes and a more severe colitis [119]. As gut bacteria are an important pathogenic factor in DSS colitis, the difference between these two opposite outcomes may be due to divergent bacterial flora colonizing mice at two research sites. Another rather ambiguous picture of D6 contribution to disease emerged in an ovalbumin-induced asthma model [120]. On one hand, D6 could scavenge some but not all of its cognate chemokines and reduced leukocyte emigration into the lungs. On the other hand, the presence of D6 resulted in increased airway hyperreactivity, by a yet unknown mechanism [120].

## D6 in human disease

Much less is known about the contribution of D6 to human diseases. D6 is expressed in several human tissues and organs and is upregulated in different inflammatory and autoimmune diseases. However, it is not clear if and how such increased levels of D6 expression may contribute to the pathogenesis. Like all ACRs, D6 is expressed in human malignancies including angiosarcomas [101] and breast cancer, where its expression levels showed positive association with a disease free survival [121]. It was shown recently that one out of four genetic variants of D6 correlates with the level of liver inflammation in hepatitis C [122]. Because the known nucleotide polymorphisms of D6 are within regulatory regions, they cannot affect protein structure and chemokine binding, but may influence D6 expression [122]. Accordingly, D6 mRNA expression was higher in patients with a mild liver disease [122]. It would be of interest to explore how these D6 polymorphisms affect the outcome in other human diseases.

## CXCR7, a multifunctional ACR for CXCL12 and CXCL11

CXCR7 was originally identified as a GPCR from a dog thyroid cDNA [123] and known for many years as an orphan receptor RDC1. It was suggested to be a chemokine receptor based on sequence homology and its genomic localization [124] and deorphanized as a receptor for CXCL12 and CXCL11 [125,126]. Despite its inclusion in the systematic chemokine receptor nomenclature, CXCR7 has DRYLSIT instead of the canonical DRYLAIV motive and functions more as an ACR than a GPCR. Accordingly, the initial findings of CXCR7 signaling and involvement in mediating cell migration [125] could not be confirmed by the subsequent studies [108,126–129]. Nevertheless, alternative CXCR7-mediated signals have been observed with the downstream cellular responses coupled to the control of cell survival and adhesion [126,130–132]. These observations may be linked to the ability of CXCR7 to recruit β-arrestin resulting in MAP-kinase activation [133–135]. In addition CXCR7 can heterodimerize with signaling CXCR4 leading to modulation of CXCL12-induced responses downstream of G_αi_ [108,128,135]. The physiological significance of such heterodimerization remains to be established. Possible signaling properties aside, the main function of CXCR7 is likely to be, in line with its ACR nature, in sequestration of CXCL12 and possibly CXCL11. This function of CXCR7 its essential in controlling the formation of CXCL12 gradients required for the optimal migration of primordial germ cells in zebrafish [127,136,137]. Internalization, sequestration and scavenging of CXCL12 and CXCL11 by CXCR7 was shown in transfectants as well as mouse heart valves and human umbilical vein endothelial cells [56]. The latter may explain a feature observed in CXCR7 deficient mice involving heart valve malformation, which causes perinatal or early postnatal lethality [108,138]. Scavenging function of CXCR7 was also reported in breast cancer cells [139]. It is not clear how this activity in cancer cells translates into tumor growth and metastasis-promoting effects exerted via CXCR7 in several experimental tumors, including breast and lung cancer [131]. In addition to tumor parenchyma, CXCR7 is also overexpressed in the tumor vasculature [131]. CXCR7 expression in vascular endothelium may have a function distinct from chemokine scavenging. Specific luminal-to-abluminal transcytosis of CXCL12 was shown to take place in bone marrow endothelial cells and attributed to be a function of CXCR4, the only known receptor for CXCL12 at the time [17]. It is possible that CXCR7, by analogy to DARC, may also be involved in chemokine transcytosis.

Additionally, CXCR7 was also described on subsets of B cells [108,140] and in T cells [125,141], findings contested following a failure to detect CXCR7 mRNA or protein in normal circulating human and murine leukocytes [142]. The latter work importantly highlights the fact that most of the commercially available anti-CXCR7 antibodies recognize unrelated molecules. However, CXCR7 expression still may be induced in bone marrow derived cells by stimuli present in specific tissues microenvironments or under pathological condition. For example CXCR7 is upregulated via Nuclear factor-kB pathway during malignant transformation of hematopoietic cells [143]. Additionally, CXCR7 expression has been described in cerebral cortex and bone osteocytes [138]. Functions of CXCR7 in these cellular contexts remain unknown.

## CCRL1, an ACR for homeostatic chemokines CCL19, CCL21 and CCL25

Chemokine (CC-motif) receptor-like 1 (CCRL1) [144] has been referred to as CCR10 [145], CCR11 [146] and recently often as CCX-CKR [145]. CCRL1 binds with high affinity homeostatic chemokines CCL19, CCL21 and CCL25 [145,147]. These ligands of CCR7 and CCR9 play pivotal roles in the establishment of functional microenvironments within primary [148,149] and secondary lymphoid organs [150]. CCRL1 has a modified DRYLAIV motif, DRYVAVT in humans and DRYWAVT in mice. Accordingly, it is unable to induce Ca-flux in transfectants [147]. Similarly to D6, it acts as chemokine scavenger causing ligand internalization and subsequent lysosomal degradation [55]. However, the efficiency of chemokine degradation via CCRL1 is hardly comparable to that by D6 [55]. Other more subtle effects may also be mediated by CCRL1. In polar cells it may function similarly to DARC [12] transporting and presenting its cognate chemokines. Presently it is unknown how homeostatic chemokines produced in the tissues are transported to the luminal endothelial cell surface [151]. Such transport across the high endothelial venules of the lymph nodes has been shown for CCL19 [152] and is likely to occur for CCL21 [103].

Absence of CCRL1 in mice leads to strong increase in blood CCL21 titers and increase in the levels of CCR7 ligands CCL19 and CCL21 in lymph nodes but not in spleen [153]. Despite this, CCRL1 deficient mice have reduced lymph node cellularity [153] and lower level steady-state DC lymph node homing [154]. This may reflect a failure of CCRL1 knockouts to establish pro-migratory chemokine patterns around lymphatics and within lymph nodes, although, desensitization of CCR7 following exposure to its ligands cannot be excluded. Conversely, transgenic overexpression of CCRL1 in epithelial cells of the embryonic thymus mitigates the homing of thymic precursors into the anlage [154]. These findings are in line with the scavenging function of CCRL1. In an experimental model of autoimmune encephalomyelitis (EAE) the parameters of the antigen-specific immunity are clearly diminished in the draining lymph nodes of CCRL1 deficient mice but are enhanced in the spleen leading to earlier onset of the disease [153]. It is not known if other immune-mediated diseases may also be modulated by CCRL1. Also, parameters and correlates of CCRL1 expression and function in human disease are meager. CCRL1 is upregulated in the bronchial lavage cells of sarcoidosis patients, epithelial cells in particular, but the effect on the disease progression is unknown [155]. Even the expression pattern of CCRL1 in normal human and murine tissues is not entirely clear. In EGFP-reporter mice the CCRL1 expression was shown to be restricted to non-hematopoietic cells including lymph node marginal sinuses, thymic epithelial cells and skin keratinocytes [154]. However, other reports indicate a much broader expression, at least at the mRNA level, including on subsets of leukocytes and in the intestine, heart and lung [144,145,147]. Increased CNS levels of CCL21 in CCRL1 deficient mice with experimental EAE [153] indicate a function of this ACR in the brain, where it is expressed in astrocytes and microglia [156]. This CNS expression pattern is shared with a new ACR, CCRL2, which is upregulated in astrocytes and microglia in response to LPS [157] and in the early onset EAE [158]. Human CCRL2 has been shown to bind CCL19 [159] and could potentially cooperate with CCRL1 in the regulation of the CCR7 axis.

## CCRL2, the new member of the ACR family

Chemokine (CC-motif) receptor-like 2 (CCRL2) is also known as L-CCR (LPS-inducible CC chemokine receptor related gene) [160], HCR (human chemokine receptor) [161] and chemokine receptor on activated macrophages (CRAM-A and CRAM-B). The latter designations reflect two alternatively spliced variants of CCRL2 resulting in molecules with different N-temini, but apparently identical properties [162]. The canonical DRYLAIV motif is changed to QRYLVFL in human and to QRYRVSF in mouse. Accordingly, most [159,163], but not all studies [162,164], describe a lack of CCRL2-mediated Ca-flux and cell migration. Nevertheless, phosphorylation of extracellular signal-regulated kinases 1 and 2 has been observed downstream of CCRL2 [165]. The exact mapping of interactions of CCRL2 with chemokine ligands remains controversial. An early study reported CCRL2 binding of CCL5 as well as several CCR2-ligands including CCL2 [164], with resulting signaling and cell migration, which, however, could not be independently confirmed [163]. Nevertheless, leukemic B cells, which carry CCRL2 but not any other cognate CCL5 receptor, increase in response to CCL5 the expression of CCRL2 on their surface. This response may be similar to D6 upregulation after exposure to cognate chemokines [97]. A more recent study showed binding of CCL19 to human CCRL2 [159] which would align this interceptor with CCRL1 in modifying the functional CCR7 axis. Based on the reported expression pattern of CCRL2, one can predict that it has very broad regulatory functions. It is expressed in many organs and tissues and by a variety of cells including astrocytes and microglia [157,158], lung bronchial epithelial cells [166], macrophages [160], T cells and hematopoietic precursors [167], neutrophils [162], mast cells [163], B-cells [165] and dendritic cells [168]. The two independently developed CCRL2 knockout models [163,168] should facilitate the efforts aimed at understanding the function of this enigmatic ACR. Data obtained in ovalbumin-induced asthma model in CCRL2 deficient mice show that CCRL2 supports the migration of antigen-laden lung DC into the mediastinal lymph nodes and this step is required for the full-blown disease manifestation [168]. Another function of CCRL2 involves its non-chemokine ligand, chemerin. CCRL2 expressed by mast cells can immobilize chemerin on cell membrane but does not internalize it [163]. It was hypothesized that CCRL2 on mast cell surface concentrates chemerin and presents it in trans-geometry to its signaling receptor ChemR23 on antigen presenting cells. This results in enhanced skin swelling and increase in leukocyte infiltrates into the lesions of passive cutaneous anaphylactic reactions induced by a low dose of antigen-specific IgE [163].

## Concluding remarks and future directions

The studies performed during the last several of years and reviewed here helped to propel ACRs from the fringes of chemokine research into its mainstream. It is clear now that ACR activities contribute to the majority of chemokine-driven *in vivo* phenomena and provide an important molecular bias to the pathophysiological processes that take us from the womb, through years of balanced wellbeing and via countless avenues of possible diseases, ultimately to the grave. Also, the early general perception of ACRs as savage chemokine decoys has been replaced by more deferential awareness and deeper understanding of their multiple and versatile functions. The recent discovery of the ACRs for homeostatic chemokines, progress in defining various functional facets of both “old” and “new” ACRs and mapping their expression in health and disease brought us much closer to deciphering “chemokinese”, the chemokine-based universal language of cell communication. Nevertheless, despite giant leaps in understanding ACRs, we are still not entirely sure about the grammatical rules of their involvement. Future studies should unravel plentiful obscure aspects of ACR biology including, but not limited, to the following issues. 1). Many but not all chemokines have been assigned a cognate ACR. The outstanding chemokines either may not require an ACR for their *in vivo* activities or their interactions with ACRs have not yet been uncovered. No comprehensive ligand mapping of ACRs has been attempted. This allows for a possibility that remaining chemokines may bind known ACRs. Alternatively, some of the orphan heptaspanning membrane receptors may be chemokine ACRs. It is clear that potential interactions of chemokines with putative ACRs cannot be unveiled using traditional methods of GPCR deorphanization, which rely on functional cell responses as readouts. Prospective binding studies of isotope- or fluorochrome-labeled chemokines to orphan heptaspanning receptors may uncover new chemokine ACRs. 2). It is not known what possible signaling pathways are initiated through ACRs and how broad is their spectrum for any individual ACR. It is also not clear if and how some of ACR-derived signals affect the availability and function of the intracellular intermediates downstream of GPCRs. Future systematic analysis of ACR interactomes may bring new understanding of the biochemical cascades unleashed by ACRs including those potentially harnessing GPCRs-mediated signals. 3). Upon their internalization, different ACRs complete dissimilar intracellular itineraries. To date it is not apparent which molecular cues allow ACRs to couple with alternative vesicular pathways. Yet unidentified functional moieties within the intracellular portions of ACRs may select putative secondary effectors and determine subsequent differential targeting into distinct endosomal compartments. Also, it is not clear how much the nature of ACR itself versus the subcellular makeup of any particular cell type affect the outcome of chemokine interactions with ACRs. Comparative functional studies of wild type and altered ACRs expressed in various host cells may answer these questions. 4). In addition to paradigmatic scavenging and transport, there may be other potential outcomes of chemokine binding by ACRs. These may include chemokine immobilization within functional microdomains of the cell membrane or nuclear targeting of chemokines. Morphological studies using high-resolution subcellular imaging may suggest such new ACRs activities. 5). Interrogating the functions of ACRs in knockout mice leaves questions open about the relative involvement of ACRs on different cells and at different sites as well as potential compensatory roles of other ACRs. The generation of conditional knockout mice, still outstanding for most ACRs, will allow selective ACR gene deletion in different organs and tissues. 6). On the whole, still very little is known about the exact contribution of ACRs to human diseases. Immunohistochemical mapping of ACR expression in human tissues has been challenged by the general lack of suitable specific antibodies recognizing ACRs in tissues. Potential discovery of polymorphic variants of ACRs with altered function and their correlations with courses and outcomes of human diseases would facilitate our understanding of different roles ACRs play in pathogenesis. This, in turn, should allow the design of new therapeutic strategies targeting ACRs for treatment of human diseases with significant chemokine contribution to their pathogenesis. Such approaches may involve, depending on the context, either interference with pathological ACR expression and function or, on the opposite, the induction and up-regulation of ACRs expression.
